# Retraction Note: microRNA-18a from M2 Macrophages Inhibits TGFBR3 to Promote Nasopharyngeal Carcinoma Progression and Tumor Growth via TGF-β Signaling Pathway

**DOI:** 10.1186/s11671-023-03811-x

**Published:** 2023-02-28

**Authors:** Ya Peng, Xiangsheng Li, Huowang Liu, Xiaowen Deng, Chang She, Chenxi Liu, Xinxing Wang, An Liu

**Affiliations:** 1 Department of Otolaryngology Head and Neck Surgery, Affiliated Changsha Hospital of Hunan Normal University, The Fourth Hospital of Changsha, Changsha, Hunan 410006 People’s Republic of China; 2 Department of Otolaryngology Head and Neck Surgery, Third Xiangya Hospital, Central South University, 138th Tongzipo Road, Yuelu District, Changsha, Hunan 410013 People’s Republic of China; 3 5th Department of Cardiology, Hunan Provincial People’s Hospital, The First Affiliated Hospital of Hunan Normal University, Changsha, Hunan 410005 People’s Republic of China; 4 Third Xiangya Hospital, Central South University, 138th Tongzipo Road, Yuelu District, Changsha, Hunan 410013 People’s Republic of China

**Retraction Note: Nanoscale Research Letters (2020) 15:196** https://doi.org/10.1186/s11671-020-03416-8

The Editors in Chief have retracted this article. After publication, concerns were raised about the following issues:

Apparent partial overlaps within both images in Fig. 1B.

Panel si-TGFBR3 in Fig. 3E appears to be similar to Fig. 4C, panel ADR (NC) in a previously published article by different authors [1, now retracted].

Panel miR-18a inhibitors in Fig. 3F seem similar to panel pcLINC00472 in Fig. 3B in a previously published paper by different authors [2] and panel CASC2 in Fig. 4c in a previously published paper by yet a different team of authors [3].

The authors did not respond to the request to clarify, as well to provide, raw data and the original ethics approval. The Editors in Chief, therefore, have lost confidence in the findings of this article. The authors did not respond to any correspondence from the Editors about this retraction.
